# Aposematic color polymorphism is a poor indicator of species boundaries in North American *Paranthrene* (Lepidoptera: Sesiidae) as evidenced by a multi-gene phylogeny

**DOI:** 10.1371/journal.pone.0312508

**Published:** 2024-11-27

**Authors:** William H. Smith III, William H. Taft, Anthony I. Cognato

**Affiliations:** Department of Entomology, Michigan State University, East Lansing, MI, United States of America; Government College University Faisalabad, PAKISTAN

## Abstract

Color polymorphism among animal species can influence speciation. Factors such as natural and sexual selection, genetic drift and gene flow contribute to the maintenance of color polymorphism within the species or spur speciation. The evolutionary and ecological mechanisms for color polymorphism are taxon specific and only a few species have been studied. A phylogeny provides an evolutionary framework to understanding the association between color polymorphism and species. *Paranthrene* species are day flying aposematic moths that mimic wasps in both appearance and behavior. The genus has several polymorphic species and some color forms were originally described as species. *Paranthrene* presents the opportunity to test for an association between color polymorphism and species boundaries. We reconstructed a phylogeny using DNA sequence from *COI*, *EF-1alpha*, and *Wingless* genes from 67 specimens representing all North American *Paranthrene* species, nine color variants, and two outgroups. Parsimony and Bayesian analyses generally agreed in topology and support values. *Paranthrene simulans* (Grote, 1881) was polyphyletic, and monophyly of *P*. *pellucida* Greenfield and Karandinos, 1979 was not recovered. *Paranthrene robiniae* Hy. Edwards, 1880 was polyphyletic and genitalic and genetic differences among the three clades supported the recognition of two new pseudocryptic species, *Paranthrene oasis* Smith, Taft and, Cognato, **new species** and *Paranthrene gilaensis* Smith, Taft and, Cognato, **new species**. *Paranthrene* color variants did not overwhelming associate with species boundaries. Of the nine color forms we examined, only two were monophyletic, had DNA sequence divergence comparable to other species, and associated with species diagnostic morphology. It is likely that genetic drift and allopatric isolation explains the fixation of color variants with species. The mechanisms maintaining color polymorphisms within *Paranthrene* species will remain unknown until experimentation concerning the degree of aposematic protection in reference to wasp models is conducted.

## Introduction

Color polymorphism is a phenomenon among animals that has been implicated as a factor in speciation [1, 2]. Selection, genetic drift and countering gene flow determine the likelihood that color variants will associate with a speciation event [2]. Specific isolating mechanisms that drive genetic divergence among color variants include allopatric isolation, assortative mating, and frequency- dependent selection. While sexual selection and natural selection in the context of heterogenous environment play an important part for several animals, each mechanism (alone or combined) can contribute to either the maintenance of polymorphism within a species or drive speciation [2]. For example, many studies of aposematic coloration, or defensive warning colors, in the context of Mullerian and Batesian mimicry demonstrate the importance of predation and the frequency of color forms [3–5]. However, both positive frequency dependent selection and male mate-choice act upon aposematic *Heliconis* (Lepidoptera) polymorphic species to drive speciation [3, 6, 7]. Discovering the role that color polymorphism plays in speciation requires observation and experimentation [2]. Most important, a phylogeny provides an evolutionary pattern of species and associated color variants which can direct experimentation [8].

**Fig 1:**
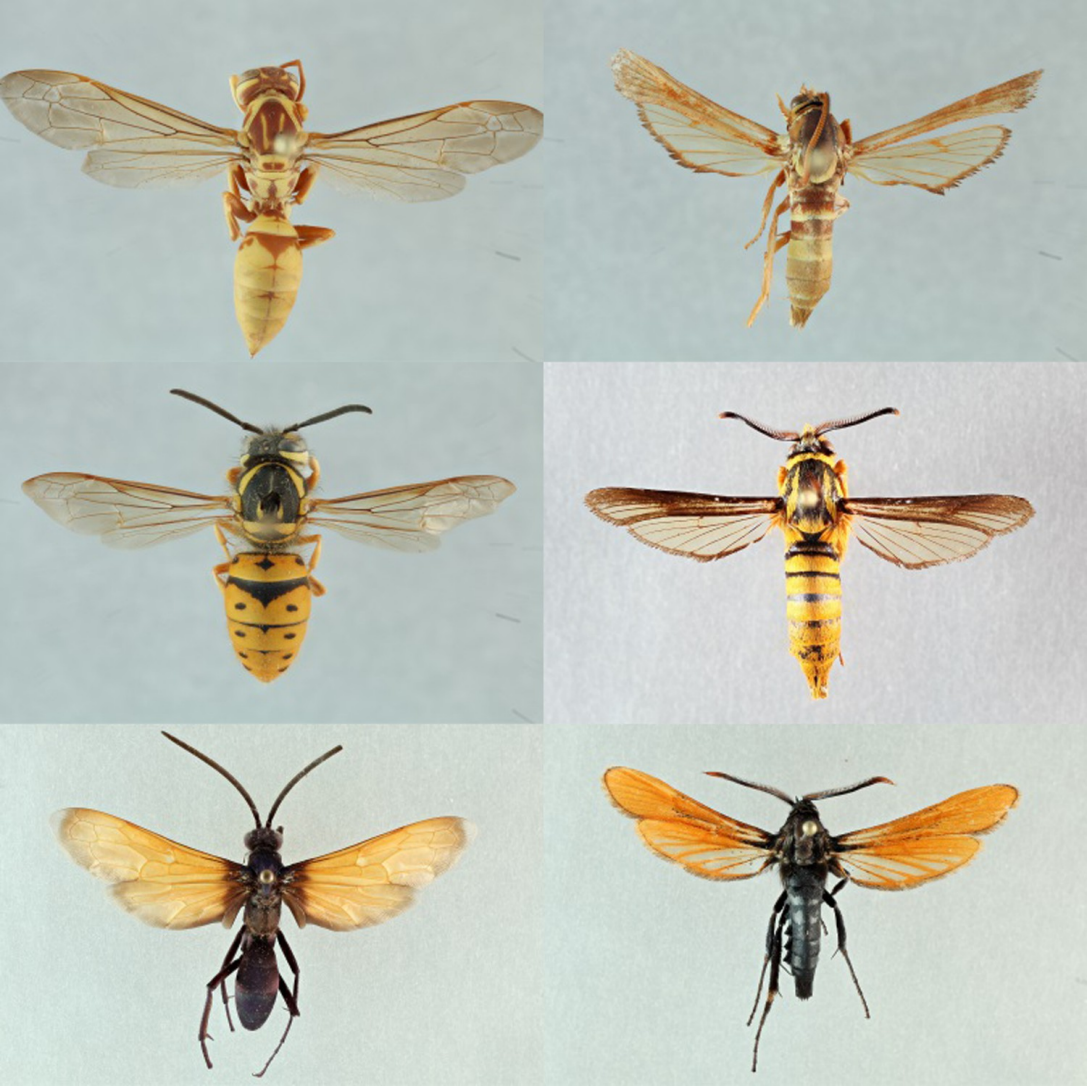
**a-c.** Potential hymenopteran models and *Paranthrene spp*. **a)** *Polistes* sp. and *Paranthrene robiniae* “palescens”, **b)** *Vespula* sp. and *Paranthrene simulans* “palmii”, **b)** *Pepsis* sp. and *Paranthrene fenestrata*.

Clearwing moths (Lepidoptera: Sesiidae) exhibit extensive aposematic color polymorphism within species [9]. All adults are diurnal mimics of wasps and bees, in both their appearance and their behavior [9]. This exhaustive mimicry is demonstrated with some species mimicking their hymenopteran model’s flight patterns and acoustics [10, 11]. As larvae, these moths bore into a variety of economically important plants, and are considered pests [12]. *Paranthrene* Hübner, 1819 typically spend two years in the larval stage and feed on oaks or willows and poplars [9, 12]. Currently, there are nine recognized *Paranthrene* species occurring in the United States, Canada, and Mexico (Baja California) along with approximately 20 species distributed in Eurasia and Africa [13–15]. Each *Paranthrene* species has two or three adult color forms, which mimic hymenopterans [9]. Likely, the color forms have converged to match various locally abundant hymenopterans (Fig 1).

**Fig 2:**
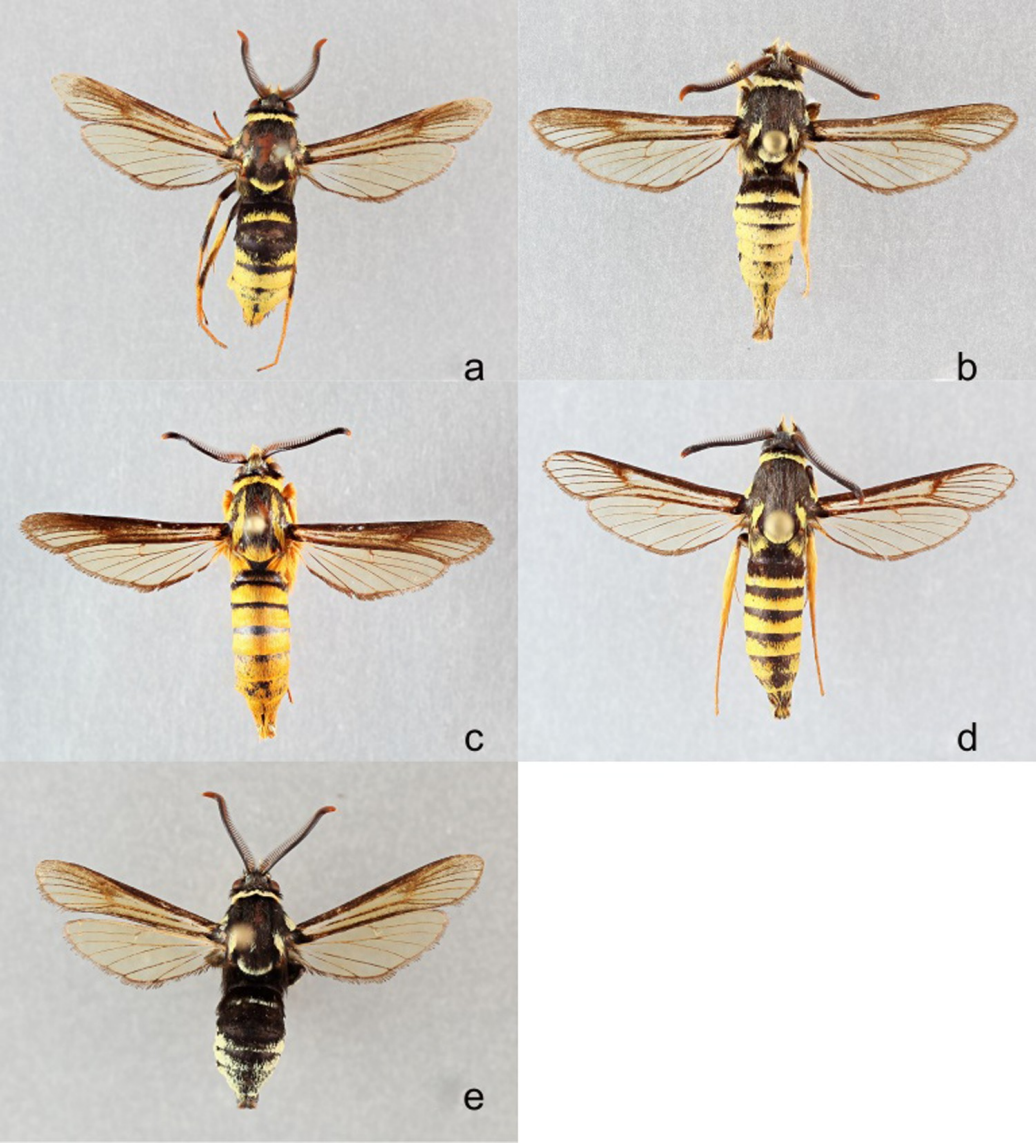
**a-e. a)** *Paranthrene simulans* “simulans” **b)** *Paranthrene simulans* “luggeri” **c)** *Paranthrene simulans* “palmii” **d)** *Paranthrene pellucida* **e)** *Paranthrene hilairemontis*.

Several *Paranthrene* species have multiple color forms. The oak borer (*Paranthrene simulans* Grote, 1881) has three unique color morphs, simulans, luggeri, and palmii forms (Fig 2A–2C). Its range includes the entire eastern United States and Canada, as far west as east Texas and Minnesota [12]. The palmii form is found in the species’ southern range and the luggeri form occurs in the western portion [9]. In the areas where simulans and palmii overlap intermediates between the forms have been observed [9]. Host plants for all populations are various oak species and the larvae feed on different parts of the tree depending on their range [12]. In the south, they feed on larger more mature trees, while in the north they are more likely to feed on saplings and small branches [16]. Prior to 1988, *Paranthrene palmii* Hy. Edwards, 1887 was considered a separate species. *Paranthrene palmii* was synonymized with *P*. *simulans* due to its similar morphology, host plants, and pheromone attraction but considered a form [9]. Likewise, luggeri was originally described in 1891 and later synonymized [17]. Despite the similarities, some behavioral and life history details have been noted between the forms. While all forms feed and have a preference for red oaks, palmii has been recorded on black oak, and never on white oaks, with the inverse observed for simulans [18]. Even their emergence is slightly different. Palmii emerges between April to June, and simulans emerges between June and July [12]. These differences, while not enough to convince Eichlin and Duckworth to recognize the palmii form as a distinct species, prompted the researchers to suggest the need for additional study of the species boundaries [9]. *Paranthrene pellucida*, Greenfield and Karandinos, 1979, is a color-form of *P*. *simulans* that is currently recognized as a species given differential response to pheromones and flight period [19]. McKern and Szalanski [20] reported intraspecific mtDNA sequence variation among Arkansian *P*. *simulans* individuals and Cognato et al. [21] showed differences among populations from Minnesota and North Carolina. Handfield and Handfield [14] demonstrated monophyly of mtDNA haplotypes that were diagnostic for a population of *P*. *simulans* from Quebec and associated with a diurnal flight period asynchronous with other *P*. *simulans*. They described this group as a new species, *P*. *hilairemontis* Handfield and Handfield 2021.

**Fig 3:**
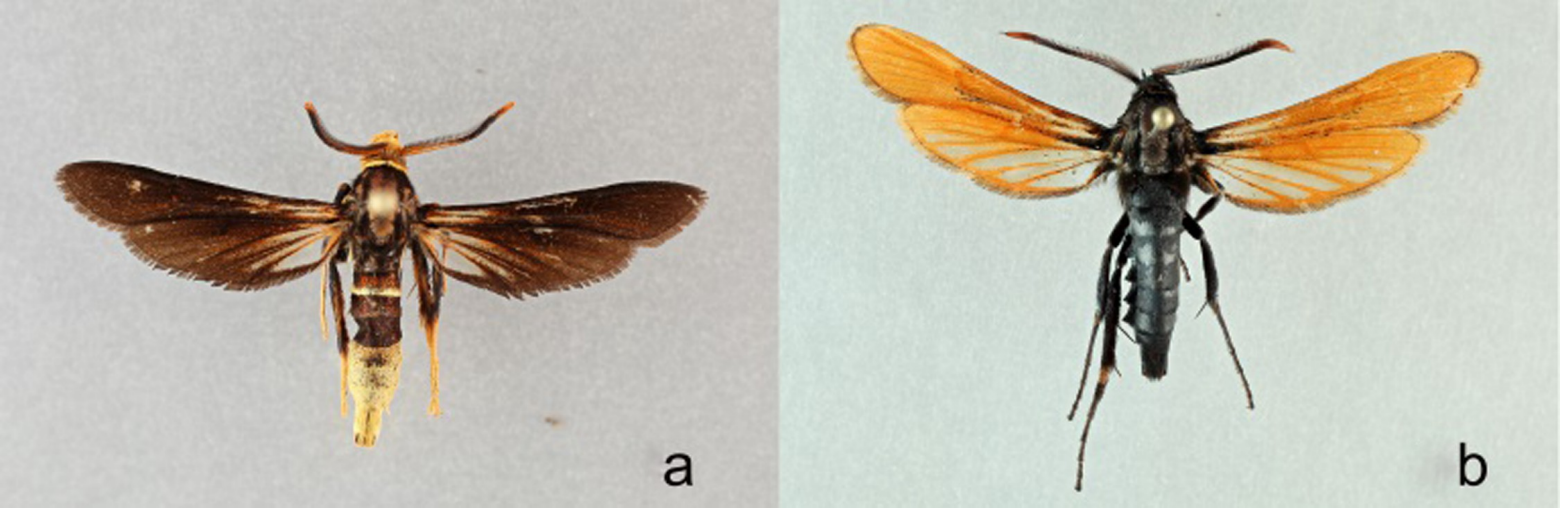
**a-b.** *Paranthrene fenestrata* **a)** yellow **b)** black.

*Paranthrene fensetrata* Barnes and Lindsey, 1922 has two distinct sympatric color morphologies (Fig 3A and 3B) with no evidence of hybrids. All specimens have been collected from high elevations in Arizona, New Mexico, Colorado, Utah, and the Mexican state of Hidalgo, otherwise, there is no life history available for this species [9].

**Fig 4:**
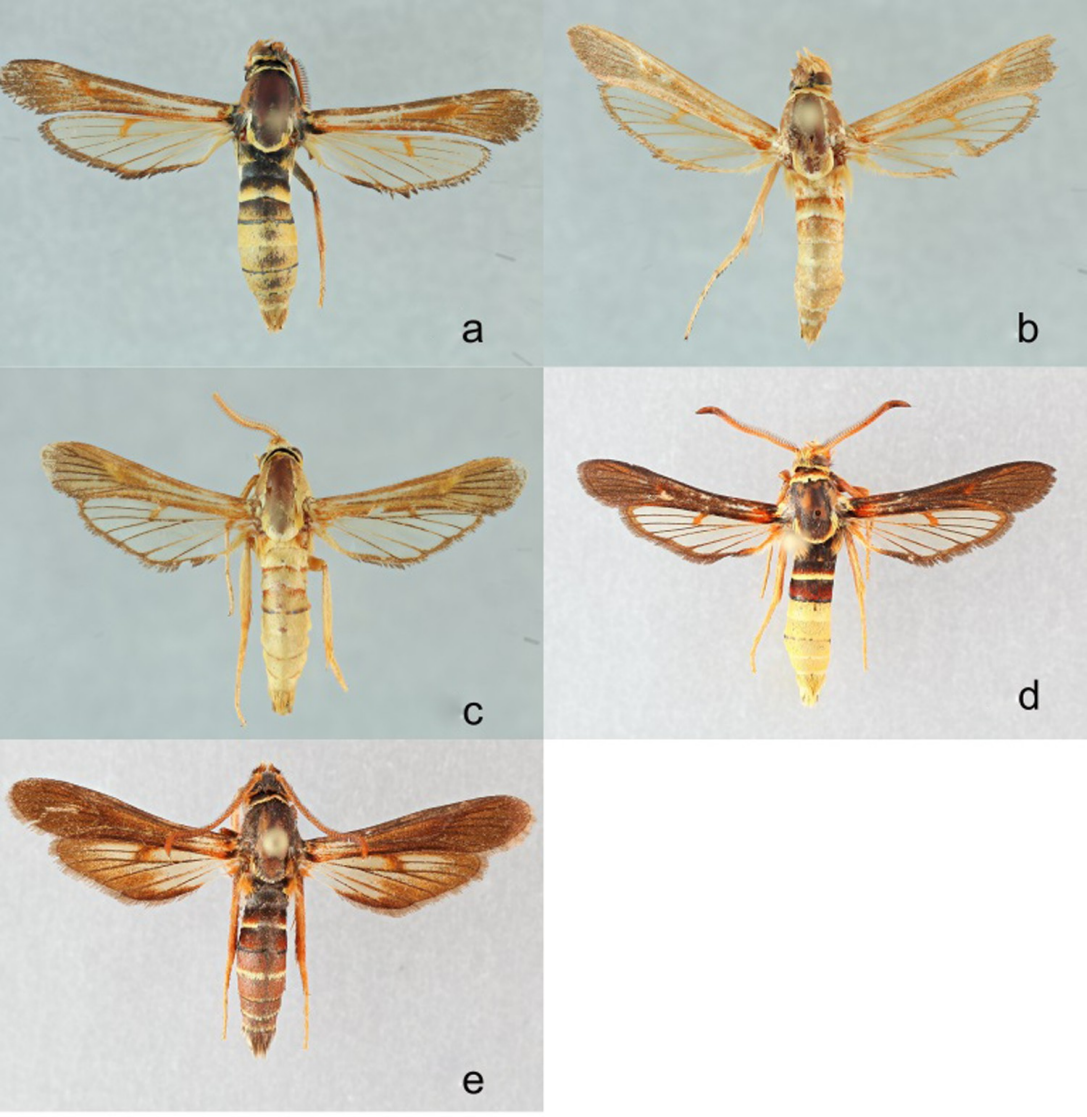
**a-e. a)** *Paranthrene robiniae* “robiniae” **b)** *Paranthrene robiniae* “palescens” **c)** *Paranthrene oasis* **d)** *Paranthrene gilaensis* **e)** *Paranthrene dollii*.

The western poplar clearwing (*Paranthrene robiniae* Edwards, 1880) is found along the Pacific Coast of the United States, the Rocky Mountains, and the provinces of British Colombia and Alberta (Canada) [16]. Its preferred host plants, poplars and willows, occur throughout western North America in isolated populations separated by other habitats non-contusive to their growth, like deserts [12, 22], There are three recognized forms, robinae, perlucida, and palescens (Fig 4A and 4B). The perlucida form occurs in Canada (British Columbia and Alberta), and the United States (the Pacific Northwest to Montana) [23]. The palescens form occurs in deserts of southern California [23] and Nevada (this study).

**Fig 5:**
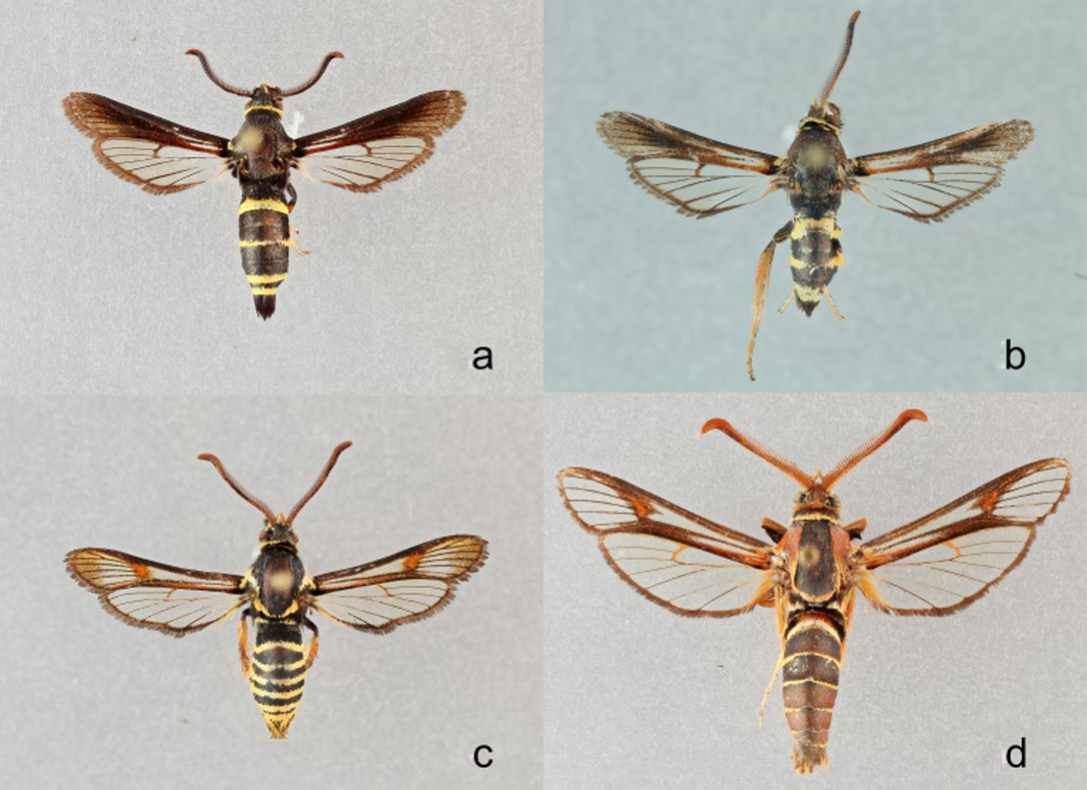
**a-d. a)** *Paranthrene tabaniformis* **b).** *Paranthrene tabaniformis* “oslari” **c)** *Paranthrene sogaardi* **d)** *Paranthrene asilipennis*.

The dusky clearwing (*Paranthrene tabaniformis* Rottemburg, 1775) (Fig 5A and 5B) is a Holarctic species [12]. Its North American range includes all of the continental United States, Alaska, and Canada, with the exception of California [24]. There are currently three recognized forms in North America, tabaniformis, denotata and oslari. Denotata is found along the Rocky Mountains from Colorado to Alaska, while oslari has been collected from Colorado and Oklahoma [23]. Recently, an unnamed color form of tabaniformis was collected and described as *P*. *sogaardi* Taft and Smith [15].

The poplar/ cottonwood clearwing borer (*Paranthrene dollii* Neumoegen, 1894) (Fig 4E) is found in the eastern United States and to the Great Plains [12]. Larvae feed the trunks and branches of poplars and willows and individual larval galleries do not intersect [9, 12]. These infestations are associated with fungal attacks [25] and beetle larvae of *Saperda* and *Cryptorhynchus* [9]. Adult flight times and generation length is unclear but potentially *P*. *dollii* has a typical two-year life span in the northern part of its range [9, 12], while southern populations may have multiple generations in a single year [12]. Outside the typical dollii form there are two other recognized color forms. The castanea form is found in the southern states as far west as the gulf coast of Texas and the fasciventris form is found in the Mid-West states surrounding Lake Michigan [23].

Phylogenetic studies of Sesiidae are few, with a majority of studies addressing the relationships among tribes and subfamilies, and only *P*. *simulans* and *P*. *tabaniformis* have been included [21, 26–29]. There are no DNA-based phylogenies of North American *Paranthrene* species, nor has any study investigated species boundaries for these species including polymorphic individuals. In this study, we reconstructed a phylogeny using all known species of North American *Paranthrene* and examined the intraspecific relationships between known color forms. We hypothesize that monophyletic groups of different color forms associate with *Paranthrene* species which would suggest that polymorphism of aposematic coloration may have a role in *Paranthrene* speciation. We found little association among color forms and species.

## Materials and methods

### Collection and identification of specimens

All but two specimens were collected over a 5-year period (year to year) using Multi-Pher #1® pheromone canister traps (Distributions Solida Inc) baited with various pheromone lures intended for different species (Table 1). Specimens were collected from public and private land where permits were not necessary. *Paranthrene* species and color forms were identified with several publications [9, 14, 15].

**Table 1:** Pheromones used for species collection.

Blend	Retailer	Target Species
ZZ 3,13 OH/EZ 3,13 OH (50:50)	Great Lakes IPM	*P*. *robiniae*
		*P*. *dollii*
		*P*. *oasis*
		*P*. *gilaensis*
		*P*. *tabaniformis*
		*P*. *sogaardi*
ZZ 3,13 A/EZ 2,13 A/Z 13 A (80: 15: 5)	Alpha Scent	*P*. *fenestrata*
		*P*. *simulans*
EZ 3,13 A/ZZ 3,13 A (4:1)	Alpha Scents	*P*. *pellucida*
ZZ 3,13 A/ZZ 2,13 OH (75:25)	Great Lakes IPM	*P*. *simulans*
		*P*. *pellucida*
EZ 2,13 A	Anglian Lepidopterist Supplies	*P*. *hilairemontis*
EZ2,13 A/Z 13 A (96:4)	Alpha Scents	*P*. *asilipennis*

### Specimen preparation and imaging

A single representative of most color forms for eight species was picked for genitalia comparisons. *Paranthrene hilairemontis* genitalia were compared to images in Handfield and Handfield [14]. The abdomen was removed at the 4th or 5th segment to access the genitals. The extracted segments were placed in individual vials filled with water and two 116 mg tablets of potassium hydroxide and allowed to sit on a hot plate set just below boiling for two hours. Softened abdominal segments were removed from the vials and teased apart under a dissecting microscope with fine-tipped forceps until genitalia were revealed. In preparation for photography, unstained genitalia were placed on a microscope slide with glycerin and spread open with fine-tipped forceps then held in place temporarily with a slide cover. Genitalia were preserved in 5 mm glass micro vials in glycerin and pinned under the associated specimen.

Specimens were photographed with a Visionary Digital Passport II system (Dun Inc., Palmyra, VA) using a Canon EOS 5D Mark II, 65.0-mm Canon Macro photo lens, two Dynalite (Union, NJ) MH2015 road flash heads, Dynalite RoadMax MP8 power pack and a Stack Shot (Cognisys, Inc, Traverse City, MI). Montage images were assembled using Zerene Stacker 1.04 and sized in Adobe Photoshop 2021 v. 22.5.1 (San Jose, CA).

### DNA sequence data and phylogenetic analyses

DNA was extracted from a metathoracic leg from 67 frozen specimens encompassing nine *Paranthrene* species representing 9 of 13 color forms along with two outgroup species (Table 2) using a Qiagen DNeasy blood and tissue kit (Hilden, Germany) following the manufacturer’s protocol. The remaining bodies were vouchered in the A. J. Cook Arthropod Research Collection. The purified DNA was used in PCR for mitochondrial *cytochrome oxidase I*, *elongation factor-1alpha*, and *wingless*. EXO-SAP-IT (USB Corp., Cleveland, OH, USA) was used to ready the PCR products for sequencing at the Michigan State University Research Technology Support Facility using Big-Dye Terminator v 1.1 (Applied Biosystems, Foster City, CA, USA) and an ABI 3730 Genetic Analyzer (Applied Biosystems). Sense and antisense strands were compiled using Sequencher (Ann Arbor, MI) to trim sequences of primer sequences, align the sequences and to create consensus sequences. Final sequences were deposited in Genbank (Table 2) and assembled in a Nexus file for a total of 1421 nucleotides (639 from *COI*, 365 from *EF-1alpha*, and 417 from *Wingless*) which included 254 parsimony-informative characters.

**Table 2:** Species, specimens, localities and Genbank numbers.

Species	Color Form	DNA voucher #	Location	Genbank # COI	Genbank # Wg	Genbank # Ef1alpha
*Vitacea admirandus*		BT 204	Medicine Park, OK	PP333558	PP338691	PP661425
*V*. *admirandus*		BT 205	Medicine Park, OK	PP333559	PP338692	PP661426
*P*. *simulans*	Unkown	BT103	Franklin, NC	PP654332	PP661526	PP661427
*P*. *simulans*	Unkown	BT 177	North Lima, OH	PP654361	PP661563	PP661471
*P*. *simulans*	simulans	BT 122	Minneapolis, MN	PP654333	PP661523	PP661428
*P*. *simulans*	simulans	BT 141	Manistee County, MI	PP654345	PP661536	PP661441
*P*. *simulans*	simulans	BT142	Manistee County, MI	PP333561	PP338694	PP661442
*P*. *simulans*	simulans	BT 143	Manistee County, MI	PP654346	PP661538	PP661443
*P*. *simulans*	simulans	BT 144	Clinton County, MI	PP654347	PP661539	PP661444
*P*. *simulans*	simulans	BT 146	Clinton County, MI	PP654349	PP661541	PP661446
*P*. *simulans*	simulans	BT 147	Clinton County, MI	PP654350	PP661542	PP661447
*P*. *simulans*	luggeri	BT 123	Minneapolis, MN	PP654334	PP661524	PP661429
*P*. *simulans*	luggeri	BT 134	Princeton, MN	PP654339	PP661530	PP661435
*P*. *simulans*	luggeri	BT 137	Princeton, MN	PP654342	PP661533	PP661438
*P*. *simulans*	luggeri	BT 138	Princeton, MN	PP654343	PP661534	PP661439
*P*. *simulans*	luggeri	BT 139	Fairfax, VA	PP654344	PP661535	PP661440
*P*. *simulans*	luggeri	BT 145	Clinton County, MI	PP654348	PP661540	PP661445
*P*. *simulans*	palmii	BT 129	Jacksonville, NC	PP333560	PP338693	PP661430
*P*. *simulans*	palmii	BT 130	Gadsden County, FL	PP654335	PP661526	PP661431
*P*. *simulans*	palmii	BT 131	Palatka, FL	PP654336	PP661527	PP661432
*P*. *simulans*	palmii	BT 132	Jacksonville, NC	PP654337	PP661528	PP661433
*P*. *simulans*	palmii	BT 133	Jacksonville, NC	PP654338	PP661529	PP661434
*P*. *simulans*	palmii	BT 135	Jacksonville, NC	PP654340	PP661531	PP661436
*P*. *simulans*	palmii	BT 136	Jacksonville, NC	PP654341	PP661532	PP661437
*P*. *simulans*	palmii	BT 172	Gainesville, FL	PP654357	PP661558	PP661466
*P*. *simulans*	palmii	BT 173	Gainesville, FL	PP654358	PP661559	PP661467
*P*. *pellucida*		BT 156	Clinton County, MI	PP654352	PP661551	PP661456
*P*. *pellucida*		BT 157	Gratiot County, Mi	PP654353	PP661552	PP661457
*P*. *pellucida*		BT 158	Gratiot County, Mi	PP654354	PP661553	PP661458
*P*. *pellucida*		BT 171	Clinton County, MI	PP654356	PP661557	PP661465
*P*. *hilairemontis*		BT 174	Quebec, Canada	PP654359	PP661560	PP661468
*P*. *fenestrata*	typical	BT 179	Emory Pass, NM	PP333575	PP338706	PP661472
*P*. *fenestrata*	typical	BT 181	Pinos Altos, NM	PP333577	PP338708	PP661474
*P*. *fenestrata*	typical	BT 182	Pinos Altos, NM	PP654362	PP661567	PP661475
*P*. *fenestrata*	yellow	BT 180	Emory Pass, NM	PP333576	PP338707	PP661473
*P*. *fenestrata*	yellow	BT 183	Emory Pass, NM	PP333578	PP338709	PP661476
*P*. *fenestrata*	yellow	BT 184	Pinos Altos, NM	PP654363	PP661569	PP661477
*P*. *fenestrata*	yellow	BT 185	Emory Pass, NM	PP654364	PP661570	PP661478
*P*. *gilaensis*	typical	BT 148	Grant County, NM	PP333562	PP661543	PP661448
*P*. *gilaensis*	typical	BT 149	Grant County, NM	PP333563	PP661544	PP661449
*P*. *gilaensis*	typical	BT 150	Grant County, NM	PP654351	PP661545	PP661450
*P*. *robiniae*	typical	BT 190	Henderson, NV	PP654366	PP661572	PP661480
*P*. *robiniae*	typical	BT 191	Henderson, NV	PP654367	PP661573	PP661481
*P*. *robiniae*	palescens	BT 188	Henderson, NV	PP654365	PP661571	PP661479
*P*. *robiniae*	palescens	BT 192	Henderson, NV	PP654368	PP661574	PP661482
*P*. *robiniae*	palescens	BT 193	Henderson, NV	PP654369	PP661575	PP661483
*P*. *oasis*		BT 194	Phoenix, AZ	PP654370	PP661576	PP661484
*P*. *oasis*		BT 195	Phoenix, AZ	PP654371	PP661577	PP661485
*P*. *tabaniformis*	typical	BT 151	McClellanville, SC	PP333564	PP338697	PP661451
*P*. *tabaniformis*	typical	BT 152	Bath Township, MI	PP333565	PP338698	PP661452
*P*. *tabaniformis*	typical	BT 153	Bath Township, MI	PP333566	PP338699	PP661453
*P*. *tabaniformis*	typical	BT 168	Ravalli County, MT	PP333571	PP338702	PP661462
*P*. *tabaniformis*	typical	BT 176	North Lima, OH	PP654360	PP661562	PP661470
*P*. *tabaniformis*	typical	BT 210	Princeton, MN	PP333580	PP338711	PP661489
*P*. *tabaniformis*	oslari	BT 214	Comanche County, OK	PP654374	PP661583	PP661491
*P*. *dollii*		BT 159	Clinton County, MI	PP654355	NA	PP661459
*P*. *dollii*		BT 160	Clinton County, MI	PP333569	NA	PP661460
*P*. *dollii*		BT 161	Clinton County, MI	PP333570	NA	PP661461
*P*. *sogaardi*		BT 154	Bath Township, MI	PP333567	PP338700	PP661454
*P*. *sogaardi*		BT 155	Bath Township, MI	PP333568	PP338701	PP661455
*P*. *sogaardi*		BT 169	Clinton County, MI	PP333572	PP338703	PP661463
*P*. *sogaardi*		BT 170	Clinton County, MI	PP333573	PP338704	PP661464
*P*. *sogaardi*		BT 211	Princeton, MN	PP333581	PP338712	PP661490
*P*. *asilipennis*		BT 175	North Lima, OH	PP333574	PP338705	PP661469
*P*. *asilipennis*		BT 197	Swanton, OH	PP654372	PP661579	PP661487
*P*. *asilipennis*		BT198	Swanton, OH	PP654373	PP661580	PP661488
*P*. *asilipennis*		BT196	Swanton, OH	PP333579	PP338710	PP661486

Phylogenetic parsimony analysis of the aligned sequences consisted of a branch and bound search using default options in PAUP v4.0a (build 168; [30]). Gaps were treated as missing data. Bootstrap values were determined with 500 pseudo-replicates each conducted by heuristic search with simple stepwise addition. Percent pairwise DNA difference was calculated as p-distance in PAUP*. In addition, Bayesian analysis under a likelihood optimality criterion was used to assess phylogenetic relationships using this dataset. Using Mr. Bayes 3.2.6 [31] two simultaneous analyses were conducted in which each gene was partitioned by codon position and a model of general time reversal + gamma + proportion of invariable sites was applied to each partition (unlinked parameters). Four Metropolis-coupled Markov chain Monte Carlo searches (one cold, three heated) were analyzed for 5 million generations. Each analysis was sampled every 100th iteration and all parameters reached stability. Bayesian posterior probabilities of clades were based on 75,002 trees—the total of both runs after a 25% burn-in.

### Species concept

We consider species as hypotheses of evolutionary lineages [32, 33]. In this study we use four criteria monophyly, differences of genitalic morphology, differences of mating behavior (pheromone blends and flight period) and DNA sequence divergence to test the hypothesis that *Paranthrene* color forms are species. Monophyly is a direct assessment of an of evolutionary lineage, differences of genitalic morphology and/or mating behavior suggest pre-mating barriers, and a 2–3% *COI* sequence divergence associates with Lepidopteran species boundaries [34, 35]. Concordance of a combination of these criteria validates species recognition.

### Nomenclatural acts

The electronic edition of this article conforms to the requirements of the amended International Code of Zoological Nomenclature, and hence the new names contained herein are available under that Code from the electronic edition of this article. This published work and the nomenclatural acts it contains have been registered in ZooBank, the online registration system for the ICZN. The ZooBank LSIDs (Life Science Identifiers) can be resolved and the associated information viewed through any standard web browser by appending the LSID to the prefix “http://zoobank.org/”. The LSID for this publication is: http://zoobank.org/urn:lsid:zoobank.org:pub:FAF78C13-980C-451E-A21C-7B30CD1B9773. The electronic edition of this work was published in a journal with an ISSN and has been archived and is available from the following digital repositories: LOCKSS.

## Results

**Fig 6:**
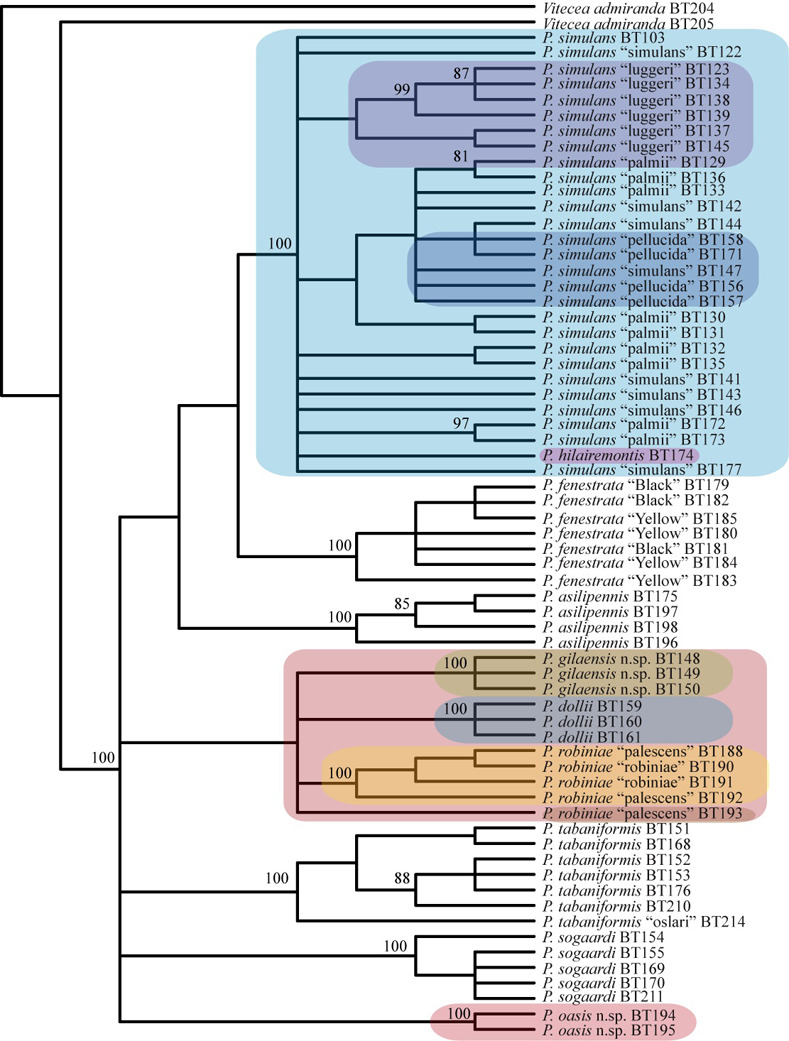
Strict consensus of 600 most parsimonious trees. Numbers above branches are bootstrap values > 80%. Color highlighted taxa are discussed in the text.

**Fig 7:**
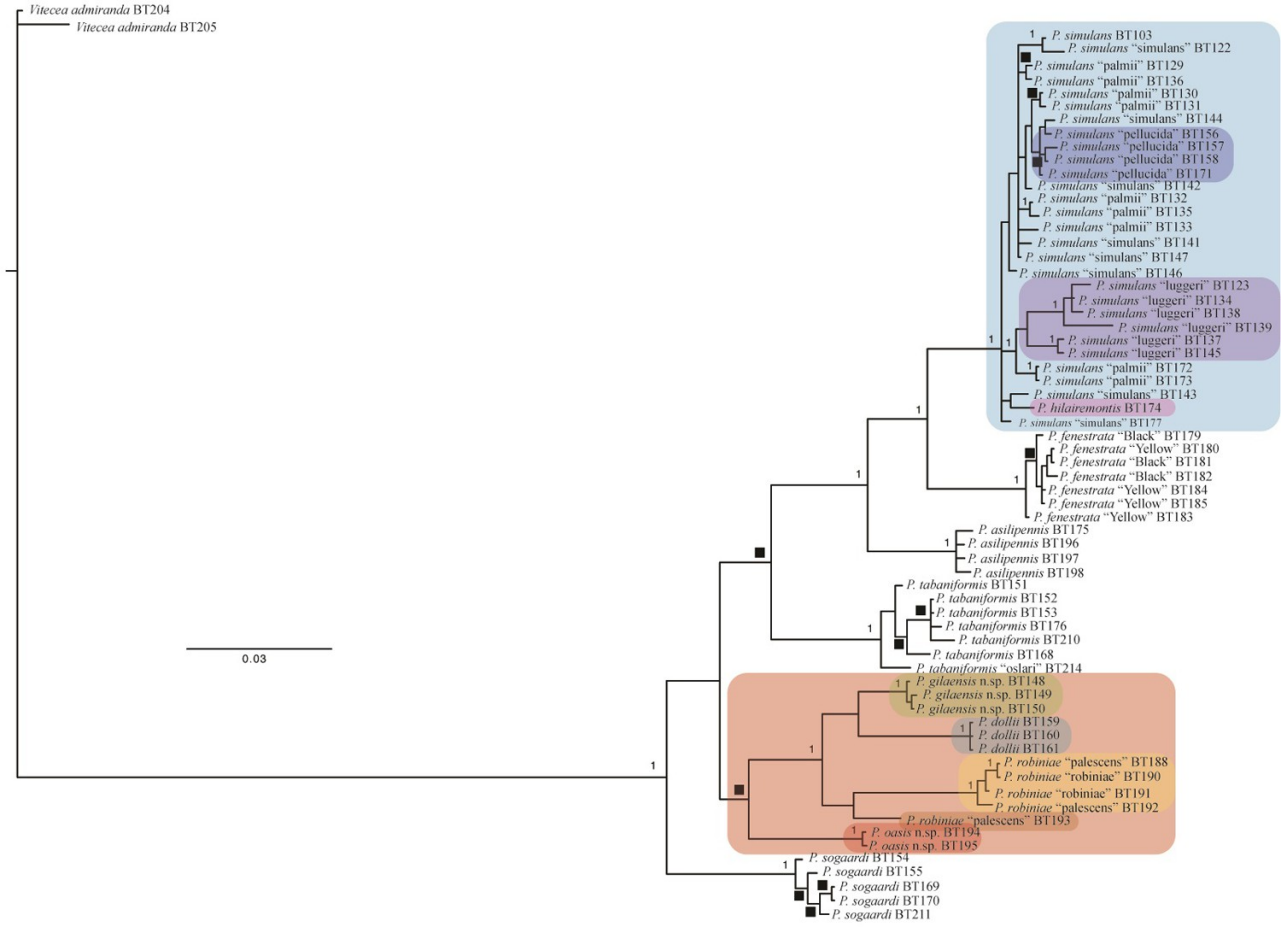
Majority-rule consensus phylogeny based on 75,002 trees resulting from the Bayesian analysis. Posterior probabilities of 1 and those between 0.9–0.99 (black square) are shown. Color highlighted taxa are discussed in the text.

The PAUP* analysis found 600 most parsimonious trees that were mostly resolved in the strict consensus of those trees (Fig 6). Intraspecific and interspecific relationships of all *P*. *simulans* forms, *P*. *pellucida* and *P*. *hilairemontis* were unresolved (Fig 6) resulting in a single clade with a 100% bootstrap value. All other clades of species except *P*. *robiniae* and *P*. *dollii* had 100% bootstrap values and values for internal nodes varied (Fig 6). The topology of the Bayesian tree was generally similar to the parsimony tree (Fig 7). Posterior probabilities (PP = 1) were high for all species clades except *P*. *pellucida*, *P*. *hilairemontis*, *P*. *robiniae* and, *P*. *dollii*. *Paranthrene pellucida* and *P*. *hilairemontis* were intermixed with *P*. *simulans*.

Within the *P*. *simulans* clade the placement of *P*. *pellucida* in both parsimony and Bayesian trees and *P*. *hilairemomtis* in the Bayesian tree rendered *P*. *simulans* as paraphyletic. *Paranthrene simulans* had an average intraspecific *COI* divergence of 1.56% and interspecific values of 1.17% and 1.27% compared to *P*. *pellucida* and *P*. *hilairemontis* respectively (Tables 3 and 4). The divergence for nuclear genes between these species was <1% and <1.35% for *EF-1alpha* and *Wg*, respectively (Tables 5 and 6). The luggeri form was the only monophyletic clade among *P*. *simulans* color forms and it rendered *P*. *simulans* paraphyletic. This clade is weakly supported in the parsimony (64% bootstrap value) and Bayesian trees (PP = 0.83) (Figs 6 and 7) and had a larger *COI* sequence divergence than any other color form (Table 3).

**Table 3:** Average percent *COI* "p" ‐ distance between *Paranthrene simulans*, *Paranthrene pellucida*, *Paranthrene hilairemontis* and, *Paranthrene fenestrata* species and color forms.

	*P*. *simulans* simulans	*P*. *simulans* luggeri	*P*. *simulans* palmii	*P*. *hilairemontis*	*P*. *pellucida*	*P*. *fenestrata* "black"	*P*. *fenestrata* "yellow"
*P*. *simulans* simulans	0.65%						
*P*. *simulans* luggerii	2.40%	2.03%					
*P*. *simulans* palmii	0.94%	2.63%	1.15%				
*P*. *hilairemontis*	0.83%	2.27%	1.11%	NA			
*P*. *pellucida*	0.55%	2.66%	0.90%	1.10%	0.26%		
*P*. *fenestrata* "black"	6.95%	7.51%	7.04%	6.36%	7.17%	0.31%	
*P*. *fenestrata* "yellow"	6.76%	7.36%	6.84%	6.26%	6.97%	0.23%	0.16%

**Table 4:** Average percent *COI* "p" ‐ distance between *Paranthrene* species.

	*P*. *asillipennis*	*P*. *dollii*	*P*. *fenestrata*	*P*. *hilairemontis*	*P*. *pellucida*	*P*. *oasis* n.sp	*P*. *gilaensis* n.sp	*P*. *robiniae*	*P*. *simulans*	*P*. *sogaardi*	*P*. *tabaniformis*	*V*. *admirandus*
*P*. *asillipennis*	0.65%											
*P*. *dollii*	8.57%	0.00%										
*P*. *fenestrata*	7.28%	10.49%	0.22%									
*P*. *hilairemontis*	6.30%	8.76%	6.31%	NA								
*P*. *pellucida*	7.00%	9.31%	7.06%	1.10%	0.26%							
*P*. *oasis* n.sp	7.39%	7.43%	8.11%	7.90%	8.45%	0.16%						
*P*. *gilaensis* n.sp	8.26%	6.26%	10.06%	9.08%	9.15%	7.43%	0.00%					
*P*. *robiniae*	8.21%	7.38%	10.20%	9.08%	9.43%	8.04%	7.62%	5.20%				
*P*. *simulans*	7.17%	9.41%	7.00%	1.27%	1.17%	8.55%	9.66%	9.55%	1.56%			
*P*. *sogaardi*	7.32%	7.67%	9.77%	8.29%	9.09%	7.56%	8.70%	8.40%	8.93%	0.47%		
*P*. *tabaniformis*	7.60%	8.03%	9.98%	7.38%	7.96%	7.49%	8.87%	8.65%	7.86%	7.46%	1.56%	
*V*. *admirandus*	13.62%	14.87%	13.90%	13.22%	13.46%	12.83%	14.95%	14.28%	13.51%	13.26%	14.14%	0.47%

**Table 5:** Average percent *EF-1alpha* "p" ‐ distance between *Paranthrene* species.

	*P*. *asillipennis*	*P*. *dollii*	*P*. *fenestrata*	*P*. *hilairemontis*	*P*. *pellucida*	*P*. *oasis* n.sp	*P*. *gilaensis* n.sp	*P*. *robiniae*	*P*. *simulans*	*P*. *sogaardi*	*P*. *tabaniformis*	*V*. *admirandus*
*P*. *asillipennis*	0.15%											
*P*. *dollii*	2.07%	0.00%										
*P*. *fenestrata*	1.27%	2.64%	0.32%									
*P*. *hilairemontis*	1.35%	3.07%	0.62%	NA								
*P*. *pellucida*	0.93%	2.26%	0.34%	0.70%	0.00%							
*P*. *oasis* n.sp	1.51%	0.58%	2.06%	1.99%	1.72%	0.00%						
*P*. *gilaensis* n.sp	1.83%	0.47%	2.35%	2.61%	2.12%	0.29%	0.19%					
*P*. *robiniae*	1.74%	0.81%	2.29%	2.33%	1.95%	0.23%	0.56%	0.22%				
*P*. *simulans*	1.28%	2.52%	0.60%	0.84%	0.32%	1.94%	2.34%	2.20%	0.48%			
*P*. *sogaardi*	2.90%	2.60%	3.18%	3.44%	2.83%	2.04%	2.40%	2.28%	3.17%	0.40%		
*P*. *tabaniformis*	2.06%	2.01%	2.55%	2.76%	2.29%	1.41%	1.80%	1.60%	2.40%	2.36%	0.42%	
*V*. *admirandus*	5.95%	5.55%	5.69%	5.31%	5.53%	5.03%	5.17%	5.11%	5.62%	5.40%	4.48%	3.44%

**Table 6:** Average percent *Wg* "p" ‐ distance between *Paranthrene* species. N/A = not available.

	*P*. *asillipennis*	*P*. *dollii*	*P*. *fenestrata*	*P*. *hilairemontis*	*P*. *pellucida*	*P*. *oasis* n.sp	*P*. *gilaensis* n.sp	*P*. *robiniae*	*P*. *simulans*	*P*. *sogaardi*	*P*. *tabaniformis*	*V*. *admirandus*
*P*. *asillipennis*	0.36%											
*P*. *dollii*	0.00%	N/A										
*P*. *fenestrata*	0.83%	N/A	0.11%									
*P*. *hilairemontis*	1.23%	N/A	0.95%	N/A								
*P*. *pellucida*	1.69%	N/A	1.19%	1.35%	0.28%							
*P*. *oasis* n.sp	3.98%	N/A	3.50%	3.89%	3.93%	0.00%						
*P*. *gilaensis* n.sp	3.37%	N/A	2.82%	3.64%	3.69%	2.72%	0.16%					
*P*. *robiniae*	3.24%	N/A	2.79%	3.60%	3.68%	2.62%	0.08%	0.00%				
*P*. *simulans*	1.47%	N/A	0.99%	0.95%	0.90%	3.76%	3.53%	3.51%	0.68%			
*P*. *sogaardi*	3.58%	N/A	3.14%	4.02%		2.47%	2.31%	2.23%	3.85%	0.29%		
*P*. *tabaniformis*	2.32%	N/A	1.85%	2.28%	2.70%	3.60%	2.89%	2.79%	2.51%	3.24%	0.20%	
*V*. *admirandus*	7.51%	N/A	7.08%	7.92%	7.34%	7.15%	6.58%	6.43%	7.71%	6.45%	6.85%	0.48%

Neither the typical black nor yellow forms of *P*. *fenstrata* were monophyletic in either tree. They resolved polyphyletic within the clade representing *P*. *fenstrata* (Figs 6 and 7). There was no DNA divergence or morphological evidence to support the recognition of these forms as species (Tables 3, 7 and 8).

**Table 7:** Average percent *EF-1alpha* "p" ‐ distance between *Paranthrene simulans*, *Paranthrene pellucida*, *Paranthrene hilairemontis* and, *Paranthrene fenestrata* species and color forms.

	*P*. *simulans* simulans	*P*. *simulans* luggeri	*P*. *simulans* palmii	*P*. *hilairemontis*	*P*. *pellucida*	*P*. *fenestrata* "black"	*P*. *fenestrata* "yellow"
*P*. *simulans* simulans	0.48%						
*P*. *simulans* luggerii	0.64%	0.45%					
*P*. *simulans* palmii	0.47%	0.50%	0.36%				
*P*. *hilairemontis*	0.64%	0.98%	0.91%	N/A			
*P*. *pellucida*	0.34%	0.35%	0.26%	0.70%	0.00%		
*P*. *fenestrata* "black"	0.69%	0.66%	0.59%	0.67%	0.39%	0.38%	
*P*. *fenestrata* "yellow"	0.56%	0.60%	0.53%	0.57%	0.29%	0.29%	0.29%

**Table 8:** Average percent *Wg* "p" ‐ distance between *Paranthrene simulans*, *Paranthrene pellucida*, *Paranthrene hilairemontis* and, *Paranthrene fenestrata* species and color forms.

	*P*. *simulans* simulans	*P*. *simulans* luggeri	*P*. *simulans* palmii	*P*. *hilairemontis*	*P*. *pellucida*	*P*. *fenestrata* "black"	*P*. *fenestrata* "yellow"
*P*. *simulans* simulans	0.43%						
*P*. *simulans* luggeri	0.89%	0.00%					
*P*. *simulans* palmii	0.52%	0.97%	0.70%				
*P*. *hilairemontis*	0.85%	0.78%	1.10%	NA			
*P*. *pellucida*	0.74%	1.04%	0.95%	1.35%	0.28%		
*P*. *fenestrata* "black"	0.75%	1.14%	1.06%	0.94%	1.18%	0.16%	
*P*. *fenestrata* "yellow"	0.77%	1.16%	1.08%	0.96%	1.20%	0.10%	0.12%

**Fig 8:**
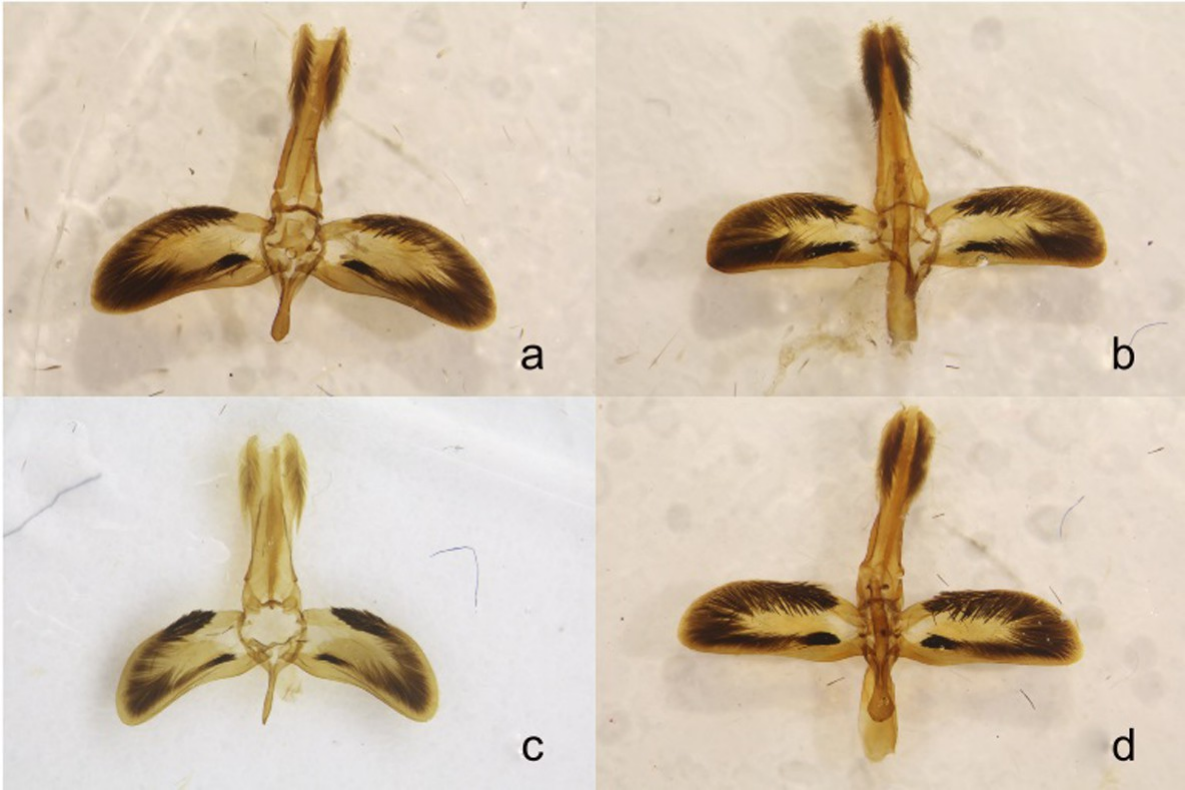
**a-d.** Genitalia **a)** *Paranthrene robiniae* **b)** *Paranthrene dollii* **c)** *Paranthrene oasis* **d)** *Paranthrene gilaensis*.

**Fig 9:**
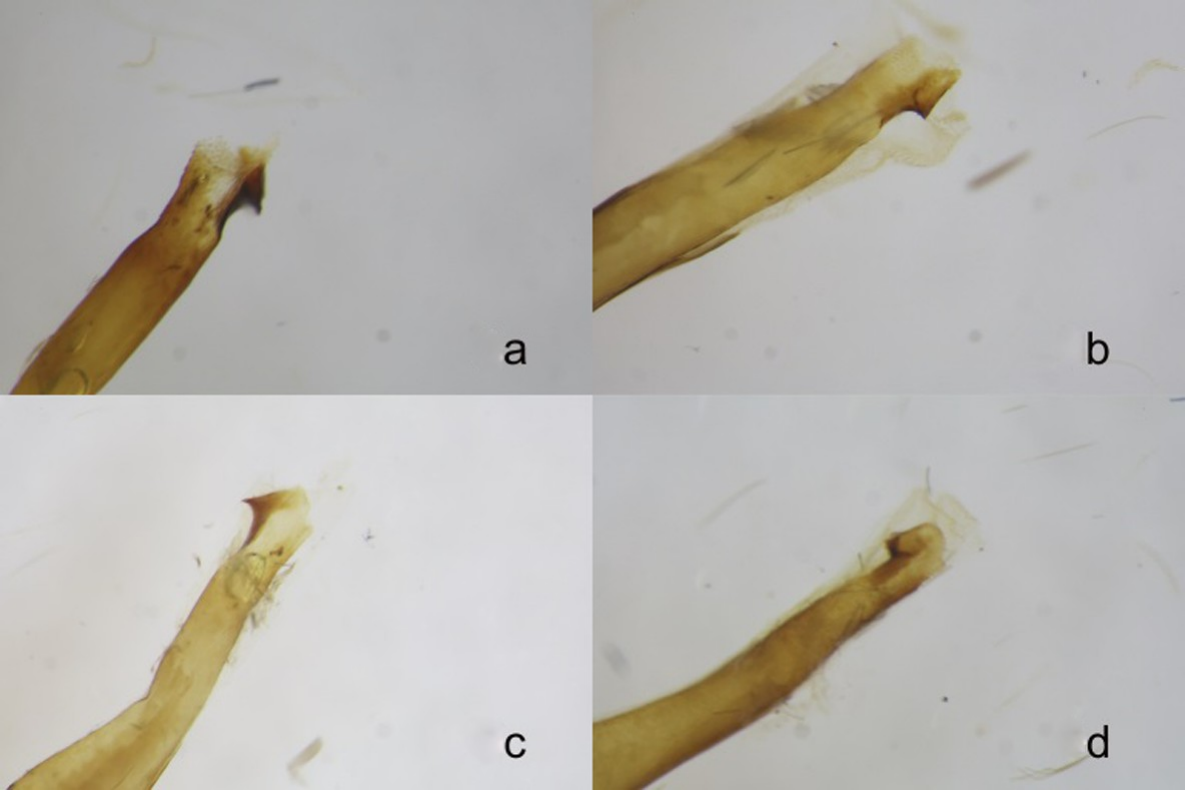
**a-d.** Adeagus spines **a)** *Paranthrene robiniae* **b)** *Paranthrene dollii* **c)** *Paranthrene oasis* **d)** *Paranthrene gilaensis*.

*Paranthrene robiniae* and *P*. *dollii* resulted in a single weakly supported clade with specimens collected at different localities monophyletic with a 100% bootstrap value (Fig 6). In the Bayesian tree, individual geographic clades of *P*. *robiniae* and *P*. *dollii* were less supported with a posterior probability ranging from 0.68–1 (Fig 7) but had similar placement as in the parsimony tree. The placement of *P*. *dollii* rendered *P*. *robiniae* paraphyletic in both trees. The palescens form *P*. *robiniae* was polyphyletic, occurring in three separate clades (Figs 6 and 7). The interspecific *COI* divergence among the three *P*. *robiniae* populations and *P*. *dollii* ranged between 6% and 9%, *EF-1alpha* divergence ranged between .19% and .86%, and *Wg* divergence ranged between 0% and 2.72%, (Tables 5, 6 and 9–11). The intraspecific *COI* divergence for each group was < 1%, except the Nevada population which was 7%. The great divergence observed in the Nevada population was caused by a single individual and removal of this single specimen decreased the average divergence to 1%. This specimen is an anomaly and requires investigation in future studies. External morphologies between the three populations showed striking similarities with little to negligible difference between the Nevada and New Mexico populations. The monophyletic Arizona and New Mexico populations had diagnostic differences of the male genitalia consistent with currently recognized species (Figs 8 and 9). The two species are described below.

**Table 9:** Average percent *COI* "p" ‐ distance between *Paranthrene robiniae*, *Paranthrene dollii*, *Paranthrene oasis* sp. nov. and, *Paranthrene gilaensis* sp. nov. species and color forms.

	*P*. *oasis* n.sp	*P*. *gilaensis* n.sp	*P*. *robiniae* typical	*P*. *robiniae* palescens	*P*. *dollii*
*P*. *oasis* n.sp	0.16%				
*P*. *gilaensis* n.sp	7.43%	0.00%			
*P*. *robiniae* typical	9.00%	7.98%	0.63%		
*P*. *robiniae* palescens	8.01%	7.25%	2.74%	5.12%	
*P*. *dollii*	7.43%	6.26%	8.29%	7.88%	0.00%

**Table 10:** Average percent *EF-1alpha* "p" ‐ distance between *Paranthrene robiniae*, *Paranthrene dollii*, *Paranthrene oasis* sp. nov. and, *Paranthrene gilaensis* sp. nov. species and color forms.

	*P*. *oasis* n.sp	*P*. *gilaensis* n.sp	*P*. *robiniae* typical	*P*. *robiniae* palescens	*P*. *dollii*
*P*. *oasis* n.sp	0.00%				
*P*. *gilaensis* n.sp	0.29%	0.19%			
*P*. *robiniae* typical	0.29%	0.67%	0.00%		
*P*. *robiniae* palescens	0.19%	0.49%	0.19%	0.29%	
*P*. *dollii*	0.58%	0.47%	0.86%	0.78%	0.00%

**Table 11:** Average percent *Wg* "p" ‐ distance between *Paranthrene robiniae*, *Paranthrene dollii*, *Paranthrene oasis* sp. nov. and, *Paranthrene gilaensis* sp. nov. species and color forms. N/A = not available.

	*P*. *oasis* n.sp	*P*. *gilaensis* n.sp	*P*. *robiniae* typical	*P*. *robiniae* palescens	*P*. *dollii*
*P*. *oasis* n.sp	0.00%				
*P*. *gilaensis* n.sp	2.72%	0.16%			
*P*. *robiniae* typical	2.62%	0.08%	0.00%		
*P*. *robiniae* palescens	2.62%	0.08%	0.00%	0.00%	
*P*. *dollii*	N/A	N/A	N/A	N/A	N/A

### Taxonomy

*Paranthrene* **Hübner**

Males of *Paranthrene* have an apical spine on the aedeagus [9].

### *Paranthrene oasis* Smith, Taft and, Cognato, new species

http://zoobank.org/urn:lsid:zoobank.org:act:668AE24C-B6C5-4315-A71D-32FD4CC99E4F.

### Type material

Holotype, male, United States: Arizona., Base and Meridian Wildlife Area, 280m ele., 33°22’37"N 112°18’24"W. 21vi2023. W, Smith Col (MSUC), second label, “DNA voucher BT195”. One paratype as previous except male genitalia dissected and with second label, “DNA voucher BT 194” (MSUC).

### Diagnosis

**Fig 10:**
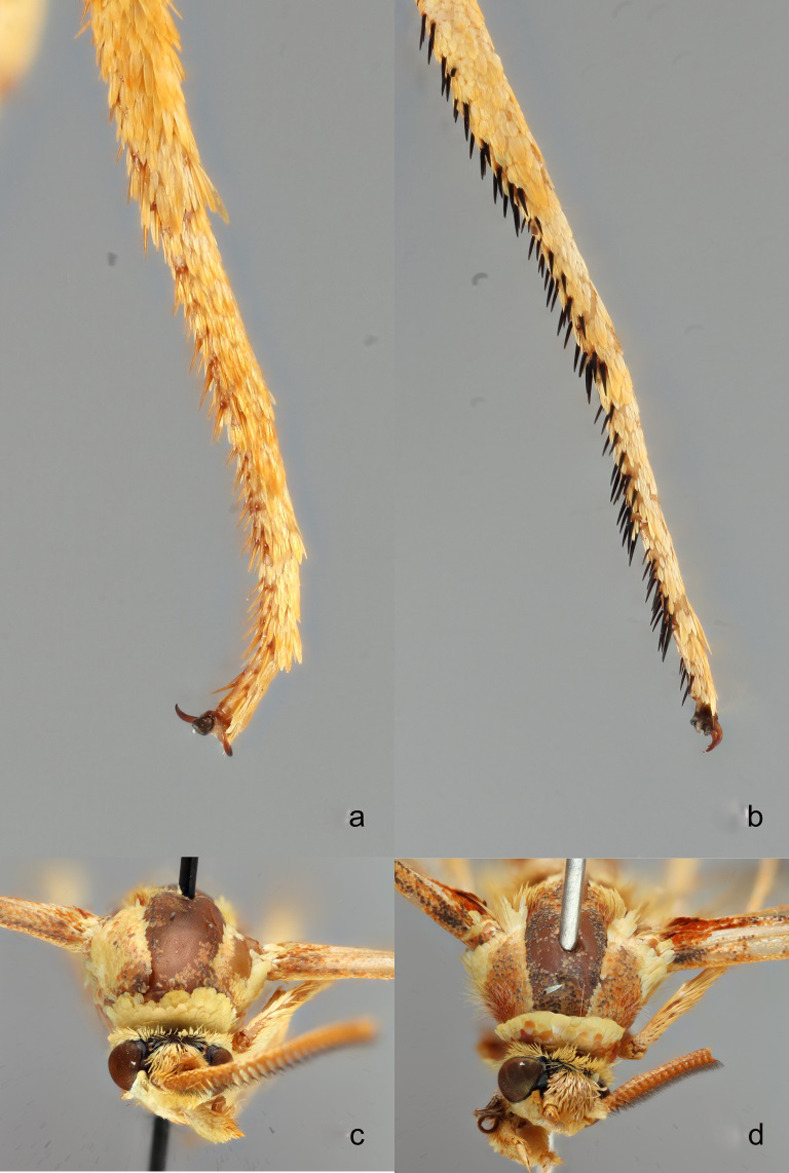
**a-d.** *Paranthrene oasis* and *P*. *robiniae* comparison **a)** *Paranthrene oasis* tarsal spines **b)** *Paranthrene robiniae* tarsal spines **c)** *Paranthrene oasis* prothoracic collar **d)** *P*. *robiniae* prothoracic collar.

**Fig 11:**
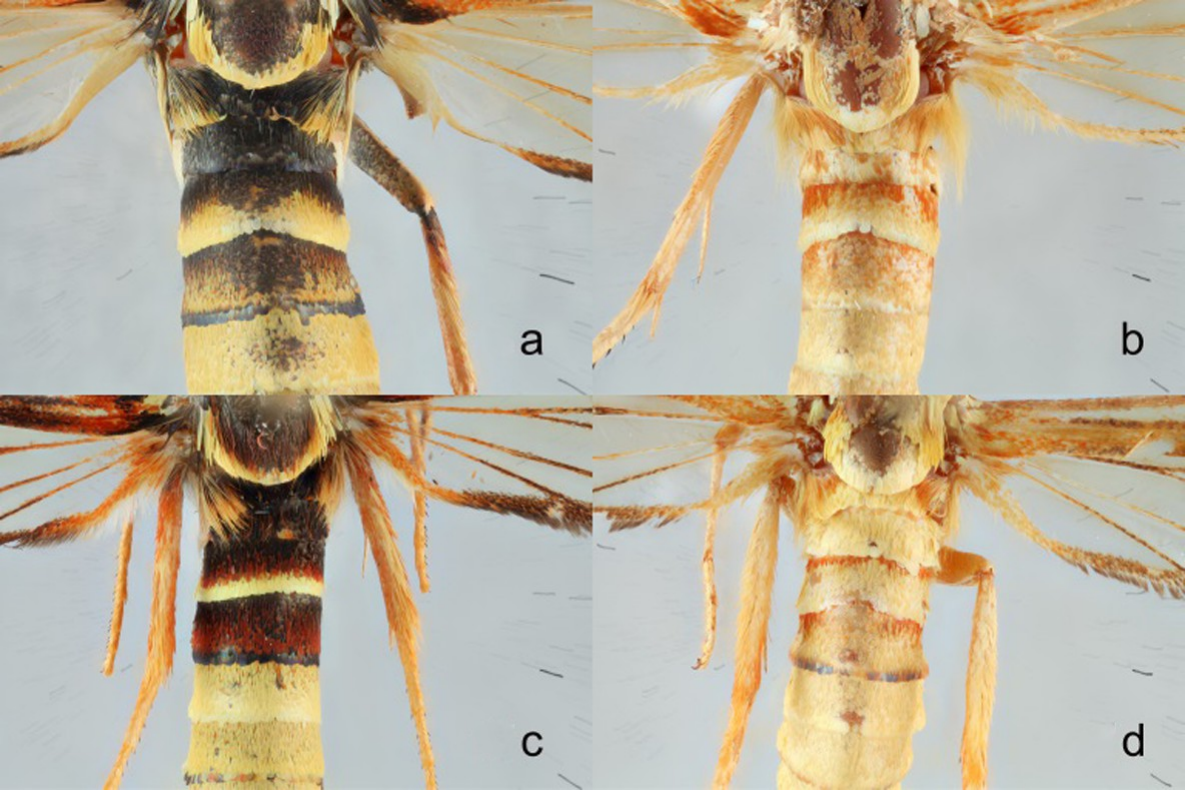
**a-d.** Abdominal color patterns **a)** *Paranthrene robiniae* “robiniae” **b)** *Paranthrene robiniae* “palescens” **c)** *Paranthrene gilaensis* **d)** *Paranthrene oasis*.

*Paranthrene oasis* is most similar to the palescens form of *P*. *robiniae*. The two species are remarkably similar in gross appearance but can be differentiated by color pattern. In *P*. *oasis* the prothoracic collar and first abdominal segment are yellow in their entirety and in *P*. *robiniae* the collar is bicolored, brown-orange proximal to the head and yellow distally, and the first abdominal segment is pigmented orange to brown (Figs 10C, 10D and 11). The tarsal spines of *P*. *oasis* are orange spines while spines of *P*. *robiniae* are black (Fig 10A and 10B). The saccus of the male genitalia, of *P*. *oasis* is slender and terminates in a point, while the saccus of *P*. *robiniae* is spatulate. (Fig 8A–8C). The aedeagus spine in *P*. *oasis* is pointed and thorn-like with few teeth, while the spine on *P*. *robiniae* is broad and ridge-like with many teeth (Fig 9).

### Description

Male (Fig 4C). Head: Vertex covered in long yellow to light orange scales; Frons light yellow; Labial palps roughened, yellow with burnt orange scales along ventral side; Haustellum coiled and longer than labial palps; Antenna burnt orange. Thorax: Prothorax covered in a solid collar of yellow scales (Fig 10C). Scutum brown and burnt orange; Scutellum brown and burnt orange bordered posteriorly with light yellow; Tegulas brown and burnt orange anteriorly transitioning to yellow posteriorly; Pluerons with broad flat burnt orange and yellow scales; Metanotum yellow, lateral anterior margins with long dense burnt orange setae; Legs primarily yellow with some burnt orange ventrally on all segments; Coxae yellow and burnt orange; Prothoracic femur concaved posteriorly and line with burnt orange setae; Mid-tibia with a single pair of ventrally apical spurs; Hind tibia with two pairs of yellow to burnt orange ventral spurs; All tarsi with ventral burnt orange spines (Fig 10A). Forewing: Mostly burnt orange to brown scales. Orange scales brightest along the base and basal portion of the inner margin; A faint orange discal spot; R, M, and C veins with dark brown scales; Brown fringe along marginal edge. Hindwing: Hyaline with dark brown fringe. Small patch of basal orange scales; Dark brown scales along all veins except R-M and M veins closing the discal cell. Abdomen (Fig 11D): Predominantly yellow with a thin anterior dorsal band of burnt orange on segment two and thin dorsal posterior bands on segments five and six. Segment three yellow and burnt orange circumferentially with a dorsally posterior band of dark brown scales; Anal tufts short and golden yellow. Male Genitalia: Saccus is shaped in a narrow triangular prism and terminates in a point. The subscaphium squares off bluntly behind the transtilla. The spine on the aedeagus is pointed and thorn like (Fig 9C).

#### Female

Unknown.

#### Host

Unknown.

#### Distribution

Known only from two specimens collected in the Base and Meridian Wildlife Area south of Phoenix Arizona near Estrella Mountain.

#### Etymology

“Oasis” used as a noun in apposition. This species is named after the environment it was found, that is, in the narrow vegetated area along the Gila River in the deserts of Arizona.

#### Remarks

*Paranthrene oasis* was collected in a trap hanging from a Fremont cottonwood *Poplus fremontii* baited with Scentry Western Poplar Clearwing Moth Lures © from Great Lakes IPM © (Table 1). There was also an abundance of Gooding’s Willows *Salix goodingii* in the habitat.

### *Paranthrene gilaensis* Smith, Taft, and Cognato, new species

http://zoobank.org/urn:lsid:zoobank.org:act:3804C23C-E988-4DD7-A565-2F0D9587E90A.

### Type material

Holotype, male, United States: New Mexico., Lake Roberts, Gila National Forest, 1846m ele., 33°01’44"N 108°09’04"W. 18vii2017. W, Taft Col. (MSUC), second label, “DNA voucher BT149”. Paratypes with same locality label. One with male genitalia dissected and second label “DNA voucher BT 148” and one with second label “DNA voucher BT 150” (2 MSUC).

### Diagnosis

*Paranthrene gilaensis* most closely resembles *P*. *robiniae*. Diagnosis is difficult due to the color polymorphism in *P*. *robiniae*. *Paranthrene gilaensis* has darker forewings and the hindwing fringe is slightly darker and thicker. On the second abdominal segment of *P*. *gilaensis* has three colored bands, anterior band is black, followed by a thin red band, and a thicker yellow posterior band (Fig 11C). In *P*. *robiniae* this middle red band is missing, although some red scales may occur but not enough to form a continuous band around the abdomen. On abdominal segment three, *P*. *gilaensis* is dorsally black and transitions laterally to red and ventrally to orange. In *P*. *robiniae* this band is black, or black and yellow for the entire circumference of the segment. Male genitalia provides unambiguous diagnostic feature. The subscaphium in *P*. *gilaensis* is bifurcated basally while in *P*. *robiniae* ends in a single point. The presence of dark scales on the hindwing differentiate *P*. *dollii* from *P*. *gilaensis* in which the dark scales are absence.

### Description

**Fig 12:**
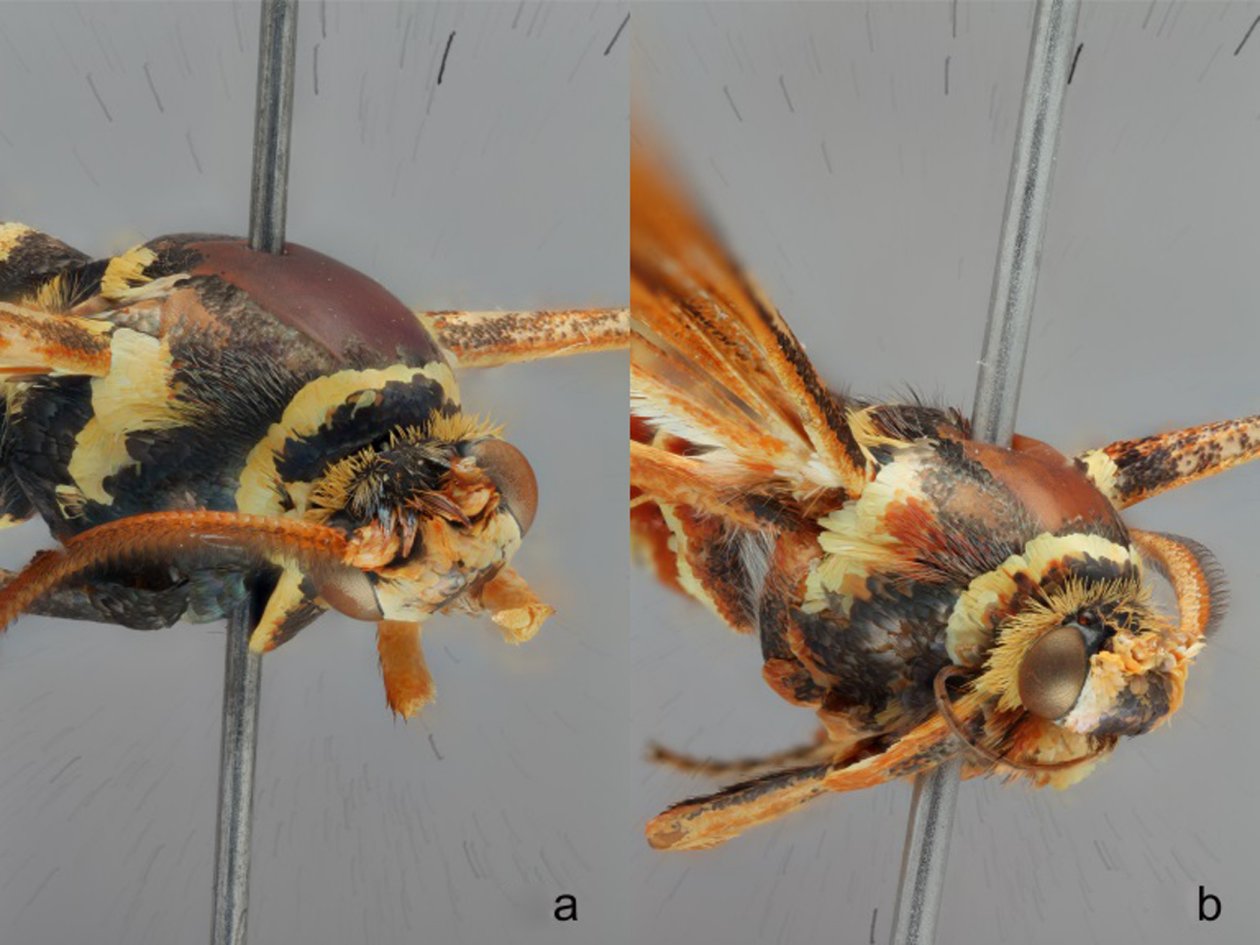
**a-b.** Tegula “shoulder” scales **a)** *Paranthrene robiniae* **b)** *Paranthrene gilaensis*.

Male (Fig 4D). Head: Vertex covered by long yellow to burnt orange scales; Frons covered in orange scales with occipital margins pale yellow, all covering a layer of smooth black scales underneath. These black scales might not be seen unless the upper layer of orange scales is rubbed off; Labial palps roughened, almost entirely light yellow and light orange except for the base which is burnt orange red and a scattering of long slender black scales; Antenna orange to orange-brown with black scales on the dorsal portion at the apical end. Thorax: Prothorax with a bicolored collar of burnt orange on the anterior margin and yellow on the posterior. The anterior orange scales cover a layer of smooth black scales similar to the frons; Scutum black with some long hair-like red scales: Scutellum black bordered posteriorly with yellow, this yellow border may be speckled with occasional red scales; Tegulas black anteriorly with many long red hairlike scales, then yellow posteriorly giving the appearance of red and yellow “shoulders” (Fig 12B); Pluerons black; Legs, Coxae black with dorsal tufts of orange scales, Femurs orange on the anterior side and black on the posterior, Mid-tibia with a single pair of ventrally apical spurs; Hind tibia with two pairs of yellow to burnt orange ventral spurs, all Tibias orange and yellow, all Tarsi orange and yellow with black spines; Forewing: Densely covered with brown and dark orange scales, Ata the base of the wing dense black scales along the anterior margin and dense red scale posteriorly. Hindwing: Hyaline with a thick dark brown fringe, bright orange scales along basal edge, all veins with brown scales except brown and burnt orange scales covering the R-M and M veins closing the discal cell. Abdomen: Predominantly yellow except for the first three segments, Segment one black, segment two with an anterior black band, then a thin red band followed by a posterior yellow band, abdominal segment three is black dorsally and transitions red laterally to yellow or orange ventrally, there is some variation with the lateral red scales forming a dorsal band between black bands, and the black scales forming a very thin circumferential anterior band; Male Genitalia: Saccus broadly spatulate and narrowed slightly at the base (Fig 8D). The subscaphium long extending behind the transtilla and terminates in a bifurcated structure.

#### Female

Unknown.

#### Host

Unknown.s

#### Distribution

Known only from Lake Roberts New Mexico, within the Gila National Forest.

#### Etymology

This species is named after the type locality in the Gila National Forest.

#### Remarks

*Paranthrene gilaensis* was collected in traps baited with Western Poplar Borer (EZ 3,13 OH/ZZ 3,13 OH) 50:50 pheromone blend.

## Discussion

Equating color form with sesiid species has been questioned in traditional taxonomic studies and often resulted in the synonymy species [9, 23]. The recognition of palescens as a color form of *P*. *robiniae*, as well as, palmii and luggeri, as a form of *P*. *simulans* is upheld in this study. The status of color-form is suggested for *P*. *pellucida* and *P*. *hilairemontis* given the lack of reciprocal monophyly with *P*. *simulans*, < 2% DNA sequence divergence for mitochondrial and nuclear genes (Tables 3–8), and morphologically similar male genitalia among these species. We, however, recognize these species as valid because of evidence for different mating behaviors among *P*. *pellucida* and *P*. *hilairemontis* and *P*. *simulans* [14, 19]. While rapid species radiation is suspected for Sesiidae [21], reciprocal monophyly and ultimately the validity of *P*. *pellucida* and *P*. *hilairemontis* will require testing with a better resolved and supported phylogeny based on genomic data. Conversely, this study and previous Sesiidae molecular phylogenetic studies have supported the recognition of color forms as species [15, 27]. However, of the nine color forms we examined three are monophyletic and only two have comparable DNA sequence divergence and associated with species diagnostic morphology (Figs 6 and 7). Thus, color pattern is not a predictable indicator for species boundaries.

Maintenance of color polymorphism among *Paranthrene* species is likely a complexity of many factors including natural selection and genetic drift [1, 2]. Color polymorphism of aposematic Lepidoptera can be maintained through selection against hybrids in narrow hybrid zones [3]. A zone of overlap occurs for the simulans and palmii forms [9, 12, 23], but this is not the case for the majority *Paranthrene* forms whose ranges are entirely sympatric. For example, the geographic ranges of the luggeri, *P*. *pellucida*, and *P*. *hilairemontis* forms are completely sympatric with the range of the simulans form [9, 14, 36]. The range of the *P*. *robiniae* palescens color form also completely overlaps with the robiniae color form [23]. Within these sympatric ranges, local abundance of particular models may provide selection at restricted geographic scales and/or for a limited time thus creating a patchwork of color forms without mating barriers [3]. Little is known concerning the distribution of *Paranthrene* color forms and model hymenopteran species or predation intensity against poor mimics of local models.

Alternatively, sexual selection may drive the diversity of color forms [2, 37]. However, sesiids use long-range pheromones to locate mates [9]. All color forms of *P*. *simulans* were collected with the same two semiochemical blends [24]. These include the semiochemicals used in the collection of the *P*. *hilairimontis* and *P*. *pellucida* forms [14, 19, 23]. Also, all three *P*. *robiniae* populations and *P*. *dollii* were collected with the same semiochemical blend (Table 1). It is unlikely that color form and pheromone type are genetically linked. Thus, sexual selection likely does not contribute to maintenance of *Paranthrene* color forms although it is unknown if color pattern is used in close-range mate recognition.

We hypothesize genetic drift is an important factor in the fixation of color forms and it is coincidental with allopatric speciation. In this study and others, species were described or validated from mountainous sky islands and oases [21, 24]. The sky islands and deserts of southwestern United States are notable for their role in speciation due to geographic isolation [38–40]. Given that much of sesiid diversity occurs in this region, it is likely that additional cryptic or pseudocryptic species exists among color polymorphic species given the apparent recent species radiation of several sesiid genera [21]. The aberrant *P*. *robiniae* palescens form specimen (BT193) may represent a cryptic species where color form has not coincided with DNA divergence.

Color pattern is a poor indicator of species boundaries in *Paranthrene*. A combination of monophyly, morphology, mating behavior, and DNA divergence reliably delimit *Paranthrene* species boundaries. These species may include populations of different color forms. Geographic barriers appear to play an important role in limiting gene flow, especially in the American Southwest. A more comprehensive survey of geographically separated populations of *Paranthrene* and of their hymenopteran models is needed to better understand the maintenance of color forms.

## References

[1] Huxley J. Morphism and Evolution. Heredity. 1955;9(1):1–52.

[2] Gray SM, McKinnon JS. Linking color polymorphism maintenance and speciation. Trends Ecol Evol. 2006;22(2):71–9. doi: 10.1016/j.tree.2006.10.005 17055107

[3] Mallet J, Barton N. Strong natural selection in a warning-color hybrid zone. Evolution. 1989;43(2):421–31. doi: 10.1111/j.1558-5646.1989.tb04237.x 28568556

[4] Sherratt TN. The evolution of Müllerian mimicry. Naturwissenschaften. 2008;95:681–95. 18542902 10.1007/s00114-008-0403-y PMC2443389

[5] Chouteau M, Arias M, Joron M. Warning signals are under positive frequencydependent selection in nature. PNAS. 2016;113(8):2164–9. doi: 10.1073/pnas.1519216113 26858416 PMC4776528

[6] Joron M, Iwasa Y. The evolution of a Müllerian mimic in a spatially distributed community. J Theor Biol. 2005;237(1):87–103. 15975598 10.1016/j.jtbi.2005.04.005

[7] Kronforst MR, Young LG, Kapan DD, McNeely C, O’Neill RJ, Gilbert LE. Linkage of butterfly mate preference and wing color preference cue at the genomic location of wingless. PNAS. 2006;103(17):6575–80. doi: 10.1073/pnas.0509685103 16611733 PMC1458925

[8] Brower A. Parallel race formation and the evolution of mimicry in *Heliconius* butterflies: A phylogenetic hypothesis from mitochondrial DNA sequences. Evolution. 1996;50(1):195–221. 28568874 10.1111/j.1558-5646.1996.tb04486.x

[9] Eichlin, TD, Duckworth, WD. Sesioidea: Sesiidae in Dominick, R.B., et al., The Moths of America North of Mexico, fasc. 5.1. Washington DC: The Wedge Entomological Foundation; 1988.

[10] Skowron Volponi MA, McLean DJ, Volponi P, Dudley R. Moving like a model: mimicry of hymenopteran flight trajectories by clearwing moths of Southeast Asian rainforest. Biol Lett. 2018;14. 10.1098/rsbl.2018.0152 PMC6012703 29720447

[11] Skowron Volponi MA, Casacci LP, Volponi P, Barbero F. Southeast Asian clearwing moths buzz like their model bees. Front Zool. 2021;18(35). doi: 10.1186/s12983-021-00419-8 34229716 PMC8262067

[12] Solomon J. Guide to Insect Borers in North American Broadleaf Trees and Shrubs. Washington DC: US Department of Agriculture; 1995. 735 p.

[13] Eichlin TD. Clearwing moths of Baja California, Mexico (Lepidoptera: Sesiidae). Trop Lepidoptera. 1992;3(2):135–50.

[14] Handfield L, Handfield N. New Species of the Genus Paranthrene Hbn., 1819 (Lepidoptera, Sesiidae, Sesiinae). J Lepidopterist Soc. 2021;75(4):252–8.

[15] Smith III WH, Taft W, Cognato AI. A new species of *Paranthrene* (Lepidoptera: Sesiidae) from the northern midwest US. Insecta Mundi. 2024; 1051:1–9.

[16] Johnson WT, Lyon HH. Insects that feed on trees and shrubs. 2nd ed. Ithica NY: Cornell University Press; 1988. 556 p.

[17] Beutenmüller W. Critical Review of the Sesiidae Found in America, North of Mexico. Harvard University: order of the Trustees, American Museum of Natural History; 1896. 38 p. (Bulletin of the American Museum of Natural History; vol. 8).

[18] Solomon J, Morris R. Clearwing Borers in Red Oaks. U.S. Forest Service Research Note SO-39. USDA Forest Service Southern Forest Experiment Station; 1966.

[19] Greenfield MD, Karandinos MG. A new species of *Paranthrene* (Lepidoptera: Sesiidae). Proceedings Entomol Soc Wash. 1979;81(3):499–504.

[20] McKern JA, Szalanski AL. Molecular diagnostics of economically important clearwing moths (Lepidoptera: Sesiidae). Fla Entomol. 2007;90(3):475–9.

[21] Cognato AI, Taft W, Osborn RK, Rubinoff D. Multi-gene phylogeny of North American clear-winged moths (Lepidoptera: Sesiidae): a foundation for future evolutionary study of a speciose mimicry complex. Cladistics. 2023;39:1–17. doi: 10.1111/cla.12515 35944148

[22] Cooke JEK, Rood S. Trees of the people: the growing science of poplars in Canada and worldwide. Can J Bot. 2007;85[12]:1103–10.

[23] Engelhardt GP. The North American Clear-wing Moths of the Family Aegeriidae. The Ohio State University: U.S. Government Printing Office; 1946. 222 p. (Bulletin (United States National Museum), United States National Museum).

[24] Taft WH, Smitley D, Snow JW. A Guide to the Clearwing Borers of the North Central United States. East Lansing: Michigan State University; 1991 Apr p. 30. (North Central Regional Publication). Report No.: 394.

[25] Cibrián Tovar D, Universidad Autónoma Chapingo, editors. Insectos forestales de México. 1. ed. español-inglés. Chapingo, Estado de México: Universidad Autónoma Chapingo; 1995. 453 p. (Publicación / Universidad Autónoma Chapingo = Publication / Universidad Autónoma Chapingo).

[26] Taft W, Cognato AI. Recognition of a new species of *Carmenta* from New Mexico supported by morphology and mitochondrial cytochrome oxidase I data (Lepidoptera: Sesiidae: Sesiinae: Synanthedonini). Zootaxa. 2017;4337(3):436–44. 29242428 10.11646/zootaxa.4337.3.8

[27] Taft W, Cognato AI, Opler PA. Phylogenetic Analysis Supports the Recognition of *Albuna beutenmulleri* Skinner as a Species Distinct from *A*. *pyramidalis* Walker (Lepidoptera: Sesiidae). J Lepidopterist Soc. 2016;70(3):211–7.

[28] Lait LA, Hebert PDN. A survey of molecular diversity and population genetic structure in North American clearwing moths (Lepidoptera: Sesiidae) using cytochrome coxidase I. PloS One. 2018;13(8). 10.1371/journal.pone.0202281 PMC6104984 30133486

[29] McKern JA, Szalanski AL, Johnson DT, Dowling APG. Molecular Phylogeny of Sesiidae (Lepidoptera) Inferred From Mitochondrial DNA Sequences. J Agric Urband Entomol. 2008;25(3):165–77.

[30] Swofford DL. PAUP: Phylogenetic Analysis Using Parsimony (and Other Methods), Version 4.0 Beta 10. Sunderland: Sinauer Associates; 2002.

[31] Ronquist F, Teslenko M, van der Mark P, Ayres DL, Darling A, Höhna S, et al. MrBayes 3.2: Efficient Bayesian Phylogenetic Inference and Model Choice Across a Large Model Space. Syst Biol. 2012 May 1;61(3):539–42. doi: 10.1093/sysbio/sys029 22357727 PMC3329765

[32] Hey J. On the failure of modern species concepts. Trends Ecol Evol. 2006 Aug;21(8):447–50. doi: 10.1016/j.tree.2006.05.011 16762447

[33] Yeates DK, Seago A, Nelson L, Cameron SL, Joseph L, Trueman JWH. Integrative taxonomy, or iterative taxonomy? Syst Entomol. 36(2):209–17.

[34] Hebert PDN, Cywinska A, Ball SL, deWaard JR. Biological identifications through DNA barcodes. Proc R Soc Lond B Biol Sci. 2003 Feb 7;270(1512):313–21. doi: 10.1098/rspb.2002.2218 12614582 PMC1691236

[35] Dincă V, Zakharov EV, Hebert PDN, Vila R. Complete DNA barcode reference library for a country’s butterfly fauna reveals high performance for temperate Europe. Proc R Soc B Biol Sci. 2011 Feb 7;278(1704):347–55. doi: 10.1098/rspb.2010.1089 20702462 PMC3013404

[36] Eichlin TD. Western Hemisphere Clearwing Moths of the Subfamily Paranthrene (Lepidoptera: Sesiidae). Entomography. 1989;6:159–212.

[37] Endler JA. Frequency-dependent predation, crypsis and aposematic coloration. Philos Trans R Soc Lond B Biol Sci. 1988;319(1196):505–23. doi: 10.1098/rstb.1988.0062 2905489

[38] Masta SE. Phylogeography of the jumping spider *Habronattus pugillis* (Araneae: Salticidae): recent vicariance of sky island populations? Evolution. 2000;54(5):1699–711. 11108597 10.1111/j.0014-3820.2000.tb00714.x

[39] Mitchell SG, Ober KA. Evolution of *Scaphinotus petersi* (Coleoptera: Carabidae) and the role of climate and geography in the Madrean sky islands of southeastern Arizona, USA. Quarternary Res. 2013;79:274–83.

[40] Edwards T, Tollis M, Hsieh P, Gutenkunst RN, Liu Z, Kusumi K, et al. Assessing models of speciation under different biogeographic scenarios; an empirical study using multi-locus and RNA-seq analyses. Ecol Evol. 2016;6(2):379–96. doi: 10.1002/ece3.1865 26843925 PMC4729248

